# Genetic Alternatives for Experimental Adaptation to Colistin in Three *Pseudomonas aeruginosa* Lineages

**DOI:** 10.3390/antibiotics13050452

**Published:** 2024-05-15

**Authors:** Igor Chebotar, Tatiana Savinova, Julia Bocharova, Dmitriy Korostin, Peter Evseev, Nikolay Mayanskiy

**Affiliations:** Laboratory of Molecular Microbiology, Center for Precision Genome Editing and Genetic Technologies for Biomedicine, Pirogov Russian National Research Medical University, Ostrovityanova 1, 117997 Moscow, Russiaivrin7@gmail.com (J.B.); d.korostin@gmail.com (D.K.); mayanskiy.nikolay@gmail.com (N.M.)

**Keywords:** *Pseudomonas aeruginosa*, antibiotic resistance, colistin, mutation, experimental evolution

## Abstract

*Pseudomonas aeruginosa* is characterized by a high adaptive potential, developing resistance in response to antimicrobial pressure. We employed a spatiotemporal evolution model to disclose the pathways of adaptation to colistin, a last-resort polymyxin antimicrobial, among three unrelated *P. aeruginosa* lineages. The *P. aeruginosa* ATCC-27833 reference strain (*Pa_ATCC*), an environmental *P. aeruginosa* isolate (*Pa_Environment*), and a clinical isolate with multiple drug resistance (*Pa_MDR*) were grown over an increasing 5-step colistin concentration gradient from 0 to 400 mg/L. *Pa_Environment* demonstrated the highest growth pace, achieving the 400 mg/L band in 15 days, whereas it took 37 and 60 days for *Pa_MDR* and *Pa_ATCC*, respectively. To identify the genome changes that occurred during adaptation to colistin, the isolates selected during the growth of the bacteria (*n* = 185) were subjected to whole genome sequencing. In total, 17 mutation variants in eight lipopolysaccharide-synthesis-associated genes were detected. *phoQ* and *lpxL*/PA0011 were affected in all three lineages, whereas changes in *pmrB* were found in *Pa_Environment* and *Pa_MDR* but not in *Pa_ATCC*. In addition, mutations were detected in 34 general metabolism genes, and each lineage developed mutations in a unique set of such genes. Thus, the three examined distinct *P. aeruginosa* strains demonstrated different capabilities and genetic pathways of colistin adaptation.

## 1. Introduction

*Pseudomonas aeruginosa* is an opportunistic pathogen with high adaptive potential, which ensures its success in clinical settings [1]. The most successful *P. aeruginosa* strains have global dissemination and belong to high-risk international epidemic clones, which are characterized by antimicrobial resistance (AMR) [2]. The evolution of AMR may be followed in vivo by comparing the isolates obtained from patients treated with antimicrobial drugs. This method was successfully used to study the phenotypic and genetic features of *P. aeruginosa* resistance in different infections, including bloodstream, lung, wound, etc., [3,4,5,6,7]. However, in such in vivo evolution studies, only a limited number of successful mutants, with the likely loss of intermediate forms, are usually available for analysis.

Another strategy for examining the evolution of AMR is based on the in vitro modeling of bacterial growth in broth media supplied with increasing antibiotic concentrations [8,9,10,11]. Consecutive re-inoculation of bacterial cultures from lower to higher antibiotic concentrations allows for the procurement of isolates with a wide resistance range. A similar procedure is used in an automated continuous culturing device (a so-called morbidostat) [12]. In these models, the experimental settings and multiple manipulations make it difficult to compare the pace of resistance evolution in different strains and observe competition among emerging clones.

In the present study, we used a modified spatiotemporal microbial evolution model [13] to monitor the evolution of resistance to the polymyxin antimicrobial, colistin, which is a last-resort antibiotic for the treatment of infections associated with multiple resistance Gram-negative bacteria. We applied this method to three *P. aeruginosa* strains of distinct origin and compared the pace of colistin resistance development. In addition, we characterized the phenotypic and genetic changes among a wide diversity of emerging clones, descendants of the three experimental strains.

## 2. Results

### 2.1. Growth Patterns and Pace of P. aeruginosa over the Colistin Concentration Gradient

**Figure 1:**
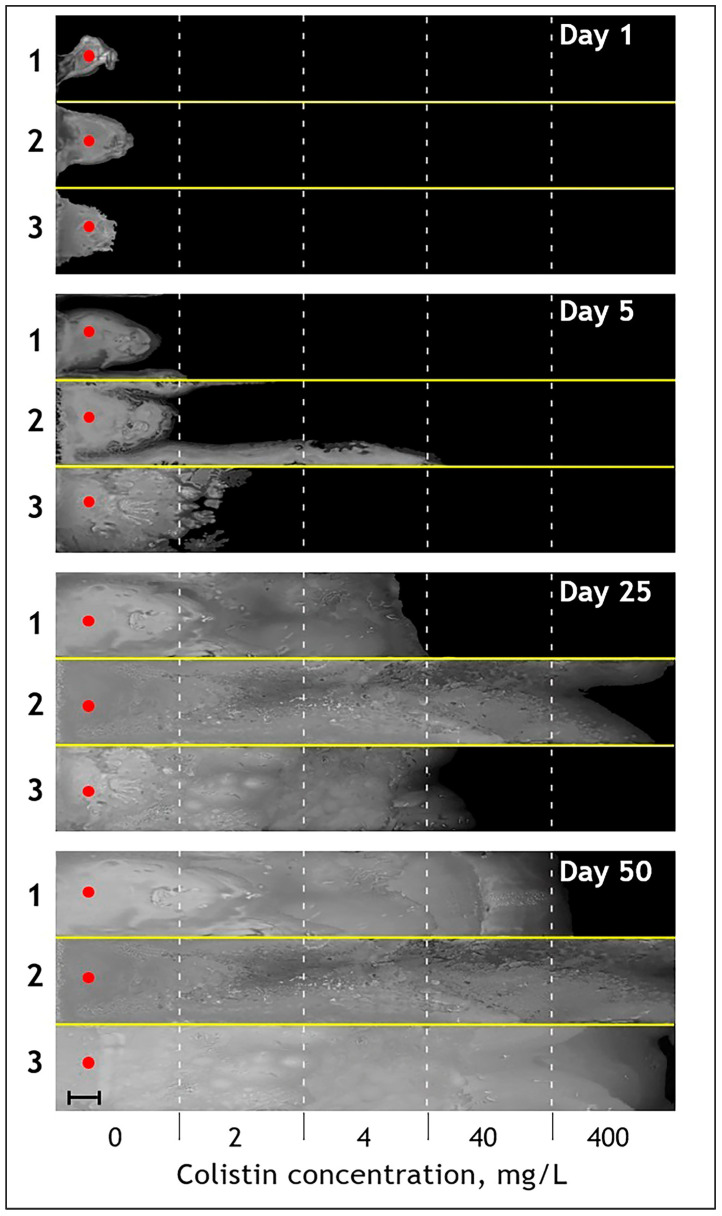
Time-lapse images of fifty-day growth patterns and pace over the colistin concentration gradient for three experimental *P. aeruginosa* lineages. Three *P. aeruginosa* strains, including *Pa_ATCC* (section 1), *Pa_Environment* (section 2), and *Pa_MDR* (section 3), were inoculated in the outermost left bands of each compartment of the experimental plate containing no colistin (red dots) and allowed to grow for indicated time. Each compartment consisted of 5 bands containing an exponential gradient of colistin from 0 to 400 mg/L. The growing bacteria appeared as a white mass against the black ink background. An image was taken from above the plate every 24 h (a movie assembled over the entire experiment is provided in Supplement). Each panel represents an unedited image obtained on the indicated day of the experiment. Bar, 40 mm.

**Figure 2:**
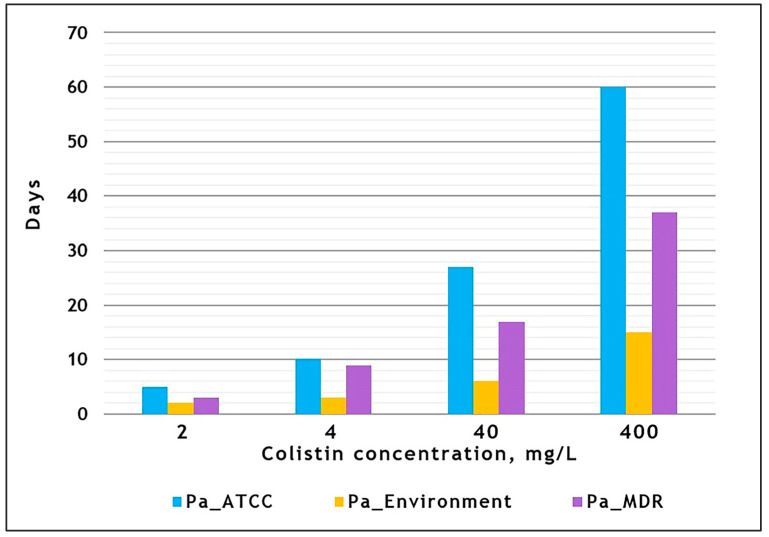
Graphical representation of the spreading dynamics over the colistin concentration gradient for three experimental *P. aeruginosa* lineages. Three experimental *P. aeruginosa* strains were grown, as described in Figure 1’s legend. The Y axis indicates the day of the experiment on which the corresponding colistin concentration band was reached. Note that smaller bars indicate faster growth, as the lineage takes less time to reach a certain colistin gradient band.

The growth of the three *P. aeruginosa* strains towards increasing colistin concentrations on the experimental plate is illustrated in Figure 1, Supplementary Figure S2, and the Supplementary Movie S1. The *Pa_Environment* strain (Figure 1, section 2) demonstrated a peculiar growth pattern, with a faster marginal spread at the beginning, whereas the remaining two strains tended towards frontal growth (Figure 1, sections 1 and 3). The marginal stream of *Pa_Environment* passed the first two colistin bands (2 and 4 mg/L) and entered the 40 mg/L band within 5 days (Figure 1, section 2, day 5; Figure 2). The frontal spread of *Pa_MDR* was slower and was dispersed across separate islands in the 2 mg/L band from day 3 to 8, reaching the 4 mg/L band on day 9 (Figure 1, section 3, day 5; Figure 2; Supplementary Movie S1). From day 12 to 13, the growth front of *Pa_MDR* became uniform (Supplementary Movie S1). *Pa_ATCC* reached the 4 mg/L band only on day 10, showing the lowest growth pace. The highest colistin concentration band of 400 mg/L was reached by the three strains at markedly different time points, varying from 15 days for *Pa_Environment* to 60 days for *Pa_ATCC* (Figure 2; Supplementary Movie S1).

In the control plate with no colistin, all three experimental strains demonstrated similar rates of propagation and occupied the entire medium surface within 10 days.

### 2.2. Genome Mutation Rate during P. aeruginosa’s Adaptation to Colistin

**Figure 3:**
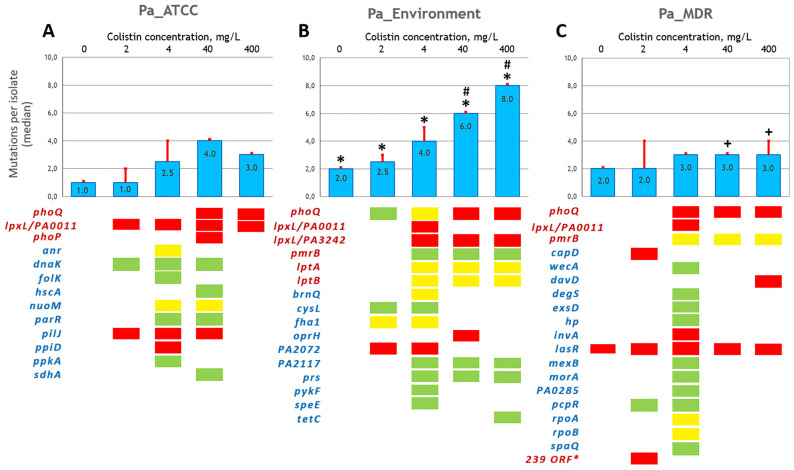
Genome mutation rate and individual gene alterations during *P. aeruginosa*’s adaptation to colistin. (**A**) *Pa_ATCC*, (**B**) *Pa_Environment*, (**C**) *Pa_MDR*. Bars show the median number with the Q3 boundary of the core genome mutations per isolate collected from the corresponding colistin concentration band, as indicated above the bars. The signs above the bars in (**B**,**C**) indicate statistically significant differences (*p* value of the Mann–Whitney U test < 0.05) in the mutation rate as follows: *, *Pa_Environment* vs. *Pa_ATCC*; #, *Pa_Environment* vs. *Pa_MDR*; +, *Pa_MDR* vs. *Pa_ATCC*. Individual affected genes are listed below the bars; red font indicates the LPS-synthesis-associated genes related to colistin resistance, blue font indicates general metabolism genes not directly related to colistin resistance. Alteration type is indicated by the rectangle fill color as follows: red, nonsense mutation/out-of-frame or large indel/gene disruption; yellow, in-frame indel; green, missense mutation. Asterisk indicates a large 262,402 bp deletion (239 ORF), which included the *galU* gene, involved in LPS synthesis.

To identify the genome changes that occurred during adaptation to colistin, the isolates selected during the spread of bacteria over the increasing colistin concentrations were subjected to WGS. In total, we sequenced 55 *Pa_ATCC*, 55 *Pa_Environment*, and 75 *Pa_MDR* isolates, including day 0 ancestral strains. *Pa_Environment* demonstrated the highest mutation rate, which consistently increased with an elevating colistin concentration from 2.5 to 7.4 mutations per isolate at 2 mg/L and 400 mg/L colistin, respectively (Figure 3B). The mutation rates in *Pa_ATCC* and *Pa_MDR* were similar and significantly lower compared to *Pa_Environment*, slightly fluctuating within the 4–400 mg/L colistin range (Figure 3A,C).

### 2.3. Core Genome Sequence Alteration Variants during P. aeruginosa’s Adaptation to Colistin

In total, 17 unique sequence alteration variants in eight lipopolysaccharide (LPS)-synthesis-associated genes were detected among the isolates of the three experimental lineages (Table 1 and Table 2, Supplementary Tables S1 and S2, Figure 3). It is noteworthy that each experimental lineage developed unique mutations that were not reproduced in the other two lineages, with the exception of the insertion ATCGCCN-1086 in *phoQ*, which was found in *Pa_ATCC* and *Pa_Environment* (Table 1). The *Pa_Environment* isolates carried the majority of these variants (11 out of 17). The *phoQ* and *lpxL*/PA0011 genes were affected in at least one descendant isolate in all three strains examined, whereas changes in the *pmrB* sequence were found in the isolates of the *Pa_Environment* and *Pa_MDR* lineage but not in the *Pa_ATCC* isolates. Mutations in *phoP* and *parR* were specific to the *Pa_ATCC* lineage, whereas mutations in *lpxL*/PA3242, *lptA*, and *lptB* were observed exclusively in the *Pa_Environment* descendants (Table 1, Figure 3).

Interestingly, diverse sequence alterations were detected in the same LPS-synthesis-associated genes among the *Pa_Environment* isolates at different time points, whereas the isolates of *Pa_ATCC* and *Pa_MDR* harbored a single mutation variant per gene (Table 1). For instance, in the *Pa_Environment* lineage, *phoQ* carried three different mutations, including a missense mutation (V260G) and two insertions, resulting in the incorrect product. Two different mutations in each gene were observed in *lpxL*/PA3242, *lpxL*/PA0011, and *lptA* (Table 1 and Table 2). Notably, the in-frame mutations in *lptA* were represented by a 6 bp insertion (ins-CCGCGC-490) and deletion (del-CCGCGC-484-489), which had an identical nucleotide sequence and were localized in the same region of *lptA*, close to nucleotide 490 (Table 1 and Table 2).

In addition, various sequence alterations were found in 34 general metabolism genes that were not directly related to colistin resistance (Table 1 and Table 2, Supplementary Tables S1 and S2, Figure 3). Remarkably, each of the studied lineages developed mutations in a unique set of genes. The *Pa_MDR* lineage possessed the largest set of affected genes (n = 14). An interesting finding was a large 262,402 bp deletion (239 ORF), which was detected in the three *Pa_MDR* isolates collected in the first week of the experiment (#A16, #A20, and #B18, Supplementary Table S2). These isolates demonstrated an increased susceptibility to amikacin and gentamycin (4- and 16-fold MIC decreases, respectively) but became more resistant to aztreonam (4-fold MIC increase). These phenotype changes could be explained by the loss of the *mexXY* operon, which was among the deleted genes. It contains efflux pump genes and the *mexZ* transcription regulator gene, which are associated with resistance to many antimicrobials, including aminoglycosides, as well as the *galU* gene, encoding uridine diphosphate glucose pyrophosphatase, involved in LPS synthesis.

### 2.4. Genetic Patterns of Adaptation to Colistin among the Three Experimental P. aeruginosa Lineages

During colistin adaptation, genetic alterations occurred in complex combinations, demonstrating diverse spatial and temporal patterns, which were *P. aeruginosa*-lineage-dependent (Figure 3, Table 2, Supplementary Tables S1 and S2). Among the *Pa_ATCC* isolates, a clone carrying mutated *lpxL*/PA0011 and *dnaK* was established at the 2 mg/L colistin band around day 10. This clone was present up to day 47, when the *dnaK* mutation was lost. Concurrently, around this time, a clone arose, which persisted until the end of the experiment, carrying the affected *phoQ* in addition to the *lpxL*/PA0011 mutation (Figure 3, Supplementary Table S1). The isolates of the *Pa_ATCC* lineage demonstrated a moderate (up to 4–8-fold) colistin MIC increase over the course of the experiment.

Two major clones were observed among the *Pa_Environment* descendants (Table 2). The evolution of one clone was associated with various alterations in *phoQ* that started on day 7, but only a 7 bp insertion mutation was fixed until the end of the experiment. In many of the isolates, *phoQ* alterations coincided with mutations in *lpxL*/PA3242, *lptA*, *lptB*, *prs*, and *hp*/PA2072. The second clone was characterized by a missense mutation in *pmrB* (L31R) occurring on day 7 in conjunction with an *hp/PA2117* mutation that appeared 1–2 days later. Both mutations persisted until the end of the experiment. The colistin MIC changes were similar among these two clones, and their maximum elevation was up to 16–32-fold. Interestingly, no isolates with simultaneous alterations in *phoQ* and *pmrB* were observed.

Limited alterations in well-established colistin resistance genes were detected in the *Pa_MDR* lineage (Supplementary Table S2). A clone with an 18 bp *pmrB* deletion occurred relatively late, on day 12, and persisted until the end of the experiment, while several *prmB* mutants carried *davD* mutations. The *pmrB* mutants of the *Pa_MDR* descendants showed a 32–64-fold increase in their colistin MICs, which were the highest among all three experimental lineages. A few *Pa_MDR* isolates carried *phoQ* and *lpxL*/PA0011 mutations, which did not coincide with *pmrB* mutations. Notably, as early as on day 2 of the experiment, among the *Pa_MDR* descendants, a prominent clone appeared carrying a nonsense mutation in the *lasR* gene (E168*), which is a transcriptional regulator involved in quorum sensing and biofilm formation. In several isolates, the *lasR* mutation coincided with alterations in other general metabolism genes not known to be directly related to colistin resistance. The *lasR* mutants demonstrated a moderate 8–16-fold increase in their colistin MICs; however, one isolate harboring a mutated *lasR* in conjunction with alterations in *phoQ* (#C50, Supplementary Table S2) showed a 64-fold colistin MIC elevation, as in the *prmB* mutants.

## 3. Discussion

The three examined *P. aeruginosa* strains of distinct origin adapted to increasing colistin concentrations at a different pace. The spread of *Pa_Environment*, a strain isolated from its natural habitat, towards increasing colistin concentrations was distinctively fast; its descendants increased in their colistin MICs by 16 times in just 7 days and reached the highest colistin concentration band on day 15. The clinical *Pa_MDR* strain and the historical strain from the ATCC collection, *Pa_ATCC*, were significantly slower in reaching the same colistin band, on days 37 and 60, respectively. These differences could not be explained by the initial variability in bacterial motility because all three lineages had similar spreading paces in the colistin-free control plate. Rather, this observation illustrated the different adaptative potential of the examined strains, although this was not formally proven statistically, which was a limitation of the present study. However, the heterogeneity of the evolutionary process in the strains of distinct origin was further emphasized by the diversity of their genomic mutations (see below).

Adaptation to colistin was accompanied by numerous alterations in the core genome, which involved (1) genes related to LPS synthesis and colistin resistance, as well as (2) various regulatory and general metabolism genes. Among the first group of genes, *phoQ* and *lpxL*/PA0011 were affected in at least one isolate for all three lineages, while *pmrB* mutations were detected in the *Pa_Environment* and *Pa_MDR* lineages. The PhoP-PhoQ and PmrA-PmrB two-component regulatory systems have been shown to contribute to bacterial tolerance and resistance to polymyxins by directly regulating genes involved in LPS modification and maintaining membrane integrity [11,14,15,16]. Our data demonstrated that *pmrB* and *phoQ* mutations were associated with a maximal increase in the colistin MICs, corroborated by the reported high colistin MICs (8 to 64 mg/L) in laboratory and clinical *P. aeruginosa pmrB* and *phoQ* mutants. The most diverse set of mutations was discovered in the *phoQ* gene, encoding a sensor histidine kinase. The *phoQ* alteration list included a number of in-frame and out-of-frame indels, as well as a missense mutation, which resulted in the V260G substitution. This amino acid substitution in PhoQ has previously been reported in clinical and experimental isolates resistant to colistin [17,18]. Interestingly, the insertion ins-ATCGCCT-1086 was identical in the *Pa_ATCC* and *Pa_Environment* descendants. This finding may indicate that the nucleotide position 1086 in the *phoQ* gene could be a hot spot targeted by the mutation process, as has been shown, for instance, for the nucleotide position 74/75 in the *pmrB* gene of colistin-resistant *Klebsiella pneumoniae* isolates [19].

The *lpxL* gene encodes for the lipid A biosynthesis lauroyl acyltransferase, involved in the production of lipid A, an outer cellular membrane component in Gram-negative bacteria. Reportedly, this gene has been associated with colistin resistance [20]. In *P. aeruginosa*, two *lpxL* homologs (PA0011 and PA3242) have been described [21], and we observed alterations in both *lpxL* variants during *P. aeruginosa*’s adaptation to colistin. In the *Pa_ATCC* lineage, a clone arose carrying *lpxL*/PA0011, which was disrupted by a 1.2 kb insertion sequence. This element was 98% identical to IS222 and belonged to the IS*3* family. The occurrence and fixation of this genetic alteration in the evolving *P. aeruginosa* population supported the idea that IS elements are not “genomic parasites” but important mediators of genome evolution and natural selection in regular or stressful environments. IS-mediated mutations have been found in many genes, including genes related to central metabolism, glucose transport, cell wall synthesis, and amino acid utilization, regulatory genes, etc. [22]. Moreover, at different stages of long-term evolution, IS-mediated alterations might exert opposite effects, producing beneficial mutations for adaptation and fitness in the early experiment stages (<60,000 bacterial generations) but eventually being detrimental for such mutants [23].

An intriguing alteration was observed in the *lptA* gene. In different *Pa_Environment* isolates, an identical 6 bp nucleotide sequence was added to (ins-CCGCGC-490) or removed from (del-CCGCGC-484-489) the same *lptA* region, close to nucleotide 490. The projection of these mutations into the amino acid sequence of LptA, a protein of the LPS transport system, indicated alterations in a region which contained Pro-Arg-Pro-Arg repeats linking two LptA domains. The truncated *lptA* generated a shortened Pro-Arg sequence, whereas the elongated *lptA* duplicated this repeat to Pro-Arg-Pro-Arg-Pro-Arg.

An interesting finding was a large 262 kb chromosomal deletion involving the *mexXY* operon detected in three *Pa_MDR* isolates between days 4 and 7 of the experiment. Large chromosomal deletions of this region have been described in *P. aeruginosa* clinical isolates and among *P. aeruginosa* chromosomal deletion model mutants, which demonstrated resistances to phages [24]. A similar deletion was found during an evolution experiment in the presence of increasing concentrations of ceftazidime or ceftazidime–avibactam [25]. Thus, our findings confirmed that genome reduction is a common strategy that supports bacteria in adapting to new environments.

Analysis of the mutation combinations revealed mutually exclusive and coexisting mutations. For instance, two separate clones emerged among the *Pa_Environment* isolates carrying either affected *pmrB* or *phoQ*, but no *pmrB/phoQ* co-mutants were detected. *phoQ* mutations coexisted with mutations in *lpxL* and *lptB*, whereas the *pmrB* mutants never carried additional mutations in the LPS-metabolism-related genes.

The second group of genes altered during *P. aeruginosa*’s adaptation to colistin included a wide variety of regulatory and metabolic genes, which have not been directly associated with colistin resistance. Among them were genes related to general metabolism (*nuoM*, *folk*, *sdhA*, *acnM*, *davD*), quorum sensing and biofilm formation, transcriptional regulation (*pilJ*, *dnaK*, *lasR*, *pcpR*, *hscA*), etc. Moreover, in each experimental *P. aeruginosa* lineage, the adaptation to colistin involved a separate set of such genes. Although their role in the development of colistin resistance remained cryptic, it is plausible to speculate that the adjustment of alternative or compensatory metabolic pathways in bacteria carrying altered LPS synthesis machinery in response to colistin pressure could be beneficial for survival. Adaptive resistance to polymyxins mediated not only by “useful” mutations but also involving alterative mechanisms, including biofilm formation, has previously been described in *P. aeruginosa* [17].

## 4. Materials and Methods

### 4.1. Bacterial Strains

Three *P. aeruginosa* strains of distinct origin were examined. These included (1) *P. aeruginosa* ATCC 27853 (*Pa_ATCC*), the reference strain for antimicrobial susceptibility testing, sequence type (ST) 155; (2) *P. aeruginosa*, isolated from the environment in 2016 (lab ID 1202), ST252, susceptible to all the tested antimicrobials (*Pa_Environment*); (3) *P. aeruginosa* with multiple drug resistance (*Pa_MDR*), isolated from an intensive care unit patient in 2013 (lab ID 39648), ST111 (international high-risk clone CC111). All the strains were susceptible to colistin (minimal inhibitory concentration (MIC) < 2 mg/L).

### 4.2. Spatiotemporal Resistance Evolution Modeling

To investigate the evolution of resistance to colistin, we modified the spatiotemporal microbial evolution model based on the spread of motile bacteria over an increasing antimicrobial gradient, proposed by Baym M. et al. [13]. The experimental plate (Supplementary Figure S1) was constructed using a clear polypropylene sheet and included three separated parallel compartments (dimension of each compartment, width × length × height, 60 × 400 × 56 mm) for a simultaneous run of the three *P. aeruginosa* strains described above. The lower part of each compartment was divided into five cells using four partitions (20 mm high), and the experimental plate was decontaminated using ultraviolet irradiation for 60 min.

The compartments were sequentially filled with three layers of medium (see below). The medium was based on LB Lennox Broth (LB, Becton Dickinson and Co., Franklin Lakes, NJ, USA) and contained 1.8%, 1.8%, and 0.23% *w/v* agar (Liofilchem srl., Abruzzi, Italy) in the bottom, middle, and top layer of the medium, respectively, as well as 5 g/L of potassium sulfate, 0.7 g/L of magnesium chloride, and 5 g/L of glycerol. To contrast the bacterial growth, 4 mL/L of black Darwi Indian Ink (The Clay & Paint Factory S.A., Wandre, Belgium) was added to the bottom and middle layers. Possible contamination was suppressed by the addition of 150 μg/L of cetyltrimethylammonium bromide (BioChemica International Inc., Melbourne, FL, USA) to the same layers of the medium. The bottom layer contained colistin (Colistin Sulfate BioChemica, ITW Reagents srl., Monza, Italy) at concentrations of 0, 2, 4, 40, and 400 mg/L.

The experimental plate was filled with the medium in the following order (Supplementary Figure S1C). First, portions of the medium containing the above concentrations of colistin were poured into the appropriate cells to form the bottom layer (19 mm high) and left to solidify. Thus, in each compartment, the bottom layer was divided into five sections with increasing colistin concentrations. Next, 20 mm of the middle medium with no colistin was layered above the bottom medium. After solidification, a 20 mm top layer of soft agar (0.23%) was added. To prevent drying, a 3 mm layer of dimethicone (polydimethylsiloxane XIAMETER™ PMX-200 Silicone Fluid 350 cSt; Dow, Midland, MI, USA) [26] was placed on the top layer.

To monitor the bacterial spreading without antimicrobial pressure, we assembled the control plate in the same way but without colistin.

Before culturing them in the constructed plate, the *P. aeruginosa* strains were adapted to movement through incubation (24 h, 35 °C) in a Petri dish with semiliquid (0.25%) LB agar, followed by re-inoculation of the marginal piece of the spreading colony into a new dish. After this procedure was repeated three times, 10 μL of the bacterial suspension (OD 0.5 McFarland) was inoculated by puncturing it into the top medium layer (Supplementary Figure S1C, layer 2) and allowed to grow at 35 °C in an air atmosphere. The spread of bacteria towards increasing colistin concentrations was video-recorded using a web camera (A4 Tech PK-910H, A-Four Tech Co. Ltd., New Taipei City, Taiwan).

For the susceptibility testing and genetic analyses, the spreading bacterial front was sampled daily at the points of advanced growth (>5 mm ahead of the front) and subcultured on Mueller–Hinton agar (Becton Dickinson and Co., Franklin Lakes, NJ, USA) for biomass accumulation; the daily cultures were stored at −82 °C before use. The topology of the selected strains is shown in Supplementary Figure S2.

### 4.3. Antimicrobial Susceptibility Testing

Colistin MICs in the range of 0.25–16 mg/L were determined using commercial plates (ComASP Colistin 0.25–16; Liofilchem srl., Abruzzi, Italy). Higher MICs were detected using broth microdilution according to EUCAST Breakpoint tables for interpretation of the MICs and zone diameters (version 14.0). A ≥4-fold colistin MIC increase was considered significant.

### 4.4. Whole Genome Sequencing (WGS)

Bacterial DNA was isolated from an overnight culture using a QIAamp DNA Mini Kit (QIAGEN, Düsseldorf, Germany). DNA libraries were prepared using an MGIEasy Universal DNA Library Prep Set (MGI Tech, Shenzhen, China) according to the manufacturer’s instructions. The DNA (300–600 ng) was fragmented using a Covaris S220 ultrasonicator (Covaris, Woburn, MA, USA). The DNA libraries were purified using magnetic particles (Agencourt AMPure XP, Beckman Coulter Life Sciences, Brea, CA, USA). The average fragment length was 250 bp. The concentrations of the DNA and the DNA libraries were measured using a Qubit Fluorometer (Thermo Fisher Scientific, Waltham, MA, USA) with the use of a dsDNA HS Assay Kit according to the manufacturer’s instructions. DNA library quality control (determination of the size distribution of the final library and confirmation of the absence of the remaining adapter dimers) was performed using a Bioanalyzer 2100 and a High Sensitivity DNA kit (Agilent Technologies, Santa Clara, CA, USA). The DNA libraries were sequenced on the DNBSEQ-G400 platform in 100 bp pair-end mode with the use of a DNBSEQ-G400RS High-throughput Sequencing Set PE100 kit (MGI Tech, China) according to the manufacturer’s instructions. A number of clones were sequenced with the help of an Oxford Nanopore Technologies MinION device according to the manufacturer’s instructions (Oxford Nanopore Technologies plc., Oxford, UK).

### 4.5. Bioinformatics

Quality control of the reads was performed using the FastQC and Trimmomatic v.0.38 software. The bacterial genomes were assembled using the SPAdes software v.3.14 [27]. For hybrid genome assembling from the MGI and Nanopore reads, the Flye, BWA, and Pilon algorithms were used [28,29]. Contamination control analysis was performed using the ContEst16S algorithm.

The QUAST 5.0 tool was used to assess the quality of the assembled genomes [30]. The genomes were annotated using the Prokka software [31]. The genome sequences obtained from the *Pa_ATCC*, *Pa_Environment*, and *Pa_MDR* strains on day 0, when the experimental plate was inoculated (*Pa_ATCC*-0, *Pa_Environment*-0, *Pa_MDR*-0), were used as a reference for the corresponding descendant clones. The breseq pipeline was used to analyze the clone genome sequencing data [32]. The clone reads were mapped onto the assembled genomes of the day 0 strains. The genetic variations presented in each clone were identified by searching for discrepancies between the aligned reads and the reference genome. Core genome mutations with a frequency in the reads of 50% or more were considered casual [33,34].

The genomes were deposited into the GenBank database (BioProject accession numbers PRJNA1013499, PRJNA1014338, and PRJNA1014908). Antimicrobial-resistance-associated gene sequences were analyzed in de novo assembled genomes using BLASTn.

### 4.6. Statistics

The statistical analysis was performed using IBM SPSS Statistics for Windows, version 27.0 (IBM Corp., Armonk, NY, USA). The mutation rates between the lineages were compared using the Kruskal–Wallis test, followed by pair-wise comparisons of the medians using the Mann–Whitney U test. The tests were considered statistically significant at *p* < 0.05.

## 5. Conclusions

In conclusion, the presented spatiotemporal model proved to be useful for dissecting the evolutionary pathways of colistin adaptation in the three *P. aeruginosa* lineages. This model allowed us to select a comprehensive set of clones, which represented all stages of the adaptation process. The examined *P. aeruginosa* strains of distinct origin demonstrated different adaptation capabilities and revealed alternative mutational patterns in the development of colistin resistance. These results underscored the importance of the initial genetic background for the development of antimicrobial resistance.

## Figures and Tables

**Table 1:** Sequence alterations in the LPS-synthesis-associated genes related to colistin resistance among three experimental lineages during adaptation to increasing colistin concentrations.

Gene	*Pa_ATCC*	*Pa_Environment*	*Pa_MDR*
*phoQ*	ins-ATCGCCT-1086	^a^ ins-ATCGCCT-1086	del-23bp-223-245
		^b^ T779→G	
		^c^ ins-CGCGAG-625	
*pmrB*	Not found	T92→G	del-18bp-298-315
*phoP*	C129→G *	Not found	Not found
*parR*	G466→A	Not found	Not found
*lpxL/PA3242*	Not found	^d^ ins-C-335	Not found
		^e^ del-35bp-525-559	
*lpxL/PA0011*	ins-IS222-517	^f^ del-A-529	del-CATG-300-303
		^g^ del-G-30	
*lptA*	Not found	^h^ ins-CCGCGC-490	Not found
		^i^ del-CCGCGC-484-489	
*lptB*	Not found	ins-GCG-27	Not found

Note. The superscript letters in the column of *Pa_Environment* designate mutation variants of one gene and match the corresponding letters in Table 2. “Not found” refers to the sequence identity of the corresponding sequence in the day 0 strain. *, premature stop-codon.

**Table 2:** Individual core gene alterations in the *Pa_Environment* lineage by colistin concentration band and time.

Isolate		Day	Colistin		*phoQ*	*pmrB*	*lpxL*/PA3242	*lpxL*/PA0011	*lptA*	*lptB*	*cysL*	*prs*	*brnQ*	*speE*	*fha1*	*hp*/PA2072	*hp*/PA2117	*tetC*	*oprH*	*pykF*
ID	Topology		Plate, mg/L	MIC, Fold Increase																
0	zero	0	0	1																
1	E7	1	0	1																
2	E8	2	0	2																
3	D9	2	0	2																
4	C8	2	0	2																
6	A9	2	0	2																
5	H26	2	4	2																
13	E10	4	0	2																
14	C11	4	2	2																
15	A13	4	2	2																
11	H30	4	4	2																
12	G21	4	4	2																
17	F25	5	4	2																
18	D11	5	2	2																
19	A18	5	2	2																
16	H31	5	40	2																
25	D23	7	4	16				^f^												
26	D13	7	2	2																
27	A20	7	2	8	^b^															
28	A22	7	4	16																
24	H33	7	40	2	^a^															
34	H34	9	40	4	^a^				^h^											
35	D25	9	4	8	^c^			^g^												
36	B22	9	4	16	^b^															
37	A27	9	4	1																
44	B27	11	4	2																
45	A29	11	4	4																
43	E32	11	40	32	^a^				i											
42	H36	11	40	32	^a^				^i^											
49	H40	13	40	1	^a^				^h^											
50	E35	13	40	16	^a^		^d^													
51	C30	13	4	1	^a^		^e^													
52	A31	13	40	16																
62	H42	16	400	2	^a^				^h^											
63	D36	16	40	16	^a^		^d^													
64	C32	16	40	16																
65	A33	16	40	2																
69	A40	17	40	16																
72	F40	18	40	1	^a^		^d^													
73	C37	18	40	2	^a^		^d^													
74	A41	18	400	16																
70	H43	18	400	1	^a^															
71	G42	18	400	8	^a^				^h^											
79	H45	20	400	2	^a^		^d^													
80	F41	20	400	2	^a^		^d^													
81	A42	20	400	8																
86	A47	21	400	16																
87	H48	24	400	16	^a^		^d^													
88	C43	24	400	16																
89	B46	24	400	16																
90	A50	24	400	8																
93	H50	27	400	16	^a^		^d^													
94	C50	27	400	16																
95	G50	28	400	16	^a^		^d^													
97	D50	28	400	16																

Note. Fifty-five isolates of the *Pa_Environment* lineage were collected from the indicated colistin concentration band (column “Plate, mg/L”) on the corresponding day of the experiment (column “Day”) and subjected to whole genome sequencing. Isolate topology is indicated according to Supplementary Figure S2. The column “MIC, fold increase” indicates the ratio of the corresponding isolate’s colistin MIC to that of the parental isolate on day 0. Gene names are highlighted in orange (LPS-synthesis-associated genes related to colistin resistance) and blue (general metabolism genes not directly related to colistin resistance). Sequence alteration type is indicated by the cell fill color (see Figure 3 legend). The letters inside the cells in the *phoQ*, *lpxL*/PA324, *lpxL*/PA0011, and *lptA* columns correspond to the mutation variant designations from Table 1 (^a^, ^b^, ^c^, ^d^, ^e^, ^f^, ^g^, ^h^, ^i^).

## Data Availability

The genomes of the isolates from the present study were deposited into the GenBank database (BioProject accession numbers PRJNA1013499, PRJNA1014338, and PRJNA1014908).

## Supplementary Materials

The following supporting information can be downloaded at: https://www.mdpi.com/article/10.3390/antibiotics13050452/s1, Supplementary Movie S1: The spread of bacteria towards increasing colistin concentrations was video-recorded using a web camera (A4 Tech PK-910H, A-Four Tech Co. Ltd., Taiwan, China). An image was taken from above the plate every 24 h, and a movie was assembled over the entire experiment. The growing bacteria appeared as a white mass against the black ink background. Figure S1: Schematic representation of the experimental plate and layers of LB medium. (A) Three-dimensional view of the experimental plate; (B) overhead view of the experimental plate; (C) cross-section of the experimental plate. Layers in (C): (1) dimethicone layer; (2) top layer of LB medium without colistin (0.23% agar); (3) middle layer of LB medium without colistin (1.8% agar); (4) bottom layer of LB medium with colistin (1.8% agar). The color corresponds to the increasing concentration of colistin in the bottom layer of medium. Red dots, inoculation points. LB, Lennox Broth. Figure S2: Topology of isolates selected for phenotypic and genetic analyses during the experimental adaptation of three *P. aeruginosa* lineages to increasing colistin concentrations. Three *P. aeruginosa* strains, including *Pa_ATCC* (upper panel), *Pa_Environment* (middle panel), and *Pa_MDR* (lower panel), were inoculated (point “zero”) into the experimental plate containing an exponential gradient of colistin from 0 to 400 mg/L (see Figure 1 for more details). Each panel was arbitrarily divided into coordinates, and collected isolates were encoded accordingly. The black vertical lines on numbers 10, 20, 30, and 40 indicate the boundaries of the colistin bands of 2, 4, 40, and 400 mg/L, respectively. Each circle indicates an isolate and its coordinates in the experimental plate, where it was collected. The white lines delineate the bacteria growth front at different time points in the experiment. Table S1: Individual gene alterations in the *Pa_ATCC* lineage by colistin concentration band and time. Table S2: Individual gene alterations in the *Pa_MDR* lineage by colistin concentration band and time. Table S3: Sequence alterations in general metabolism genes not directly related to colistin resistance among three experimental lineages during adaptation to increasing colistin concentrations.
