# Supplementary material for: Genetic Alternatives for Experimental Adaptation to Colistin in Three Pseudomonas aeruginosa Lineages
Source: Antibiotics (Basel). 2024 May 15;13(5):452. doi: 10.3390/antibiotics13050452 (PMC11117860; doi:10.3390/antibiotics13050452)
Supplement: Supplementary file 1 [file antibiotics-13-00452-s001.zip › Supplementary Figures S1 and S2.pdf]

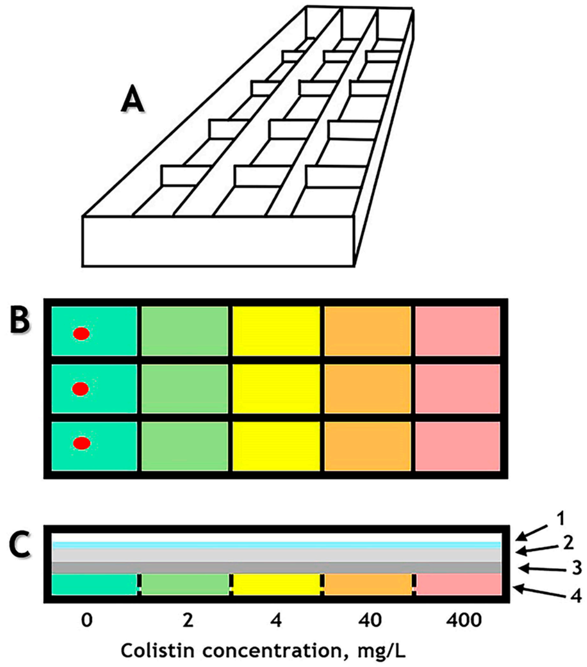

Figure S1. Schematic representation of the experimental plate and layers of LB medium. (**A**) Three-dimensional view of the experimental plate; (**B**) overhead view of the experimental plate; (**C**) cross-section of the experimental plate. Layers in (**C**): (1) dimethicone layer; (2) top layer of LB medium without colistin (0.23% agar); (3) middle layer of LB medium without colistin (1.8% agar); (4) bottom layer of LB medium with colistin (1.8% agar). The color corresponds to the increasing concentration of colistin in the bottom layer of medium. Red dots, inoculation points. LB, Lennox Broth.

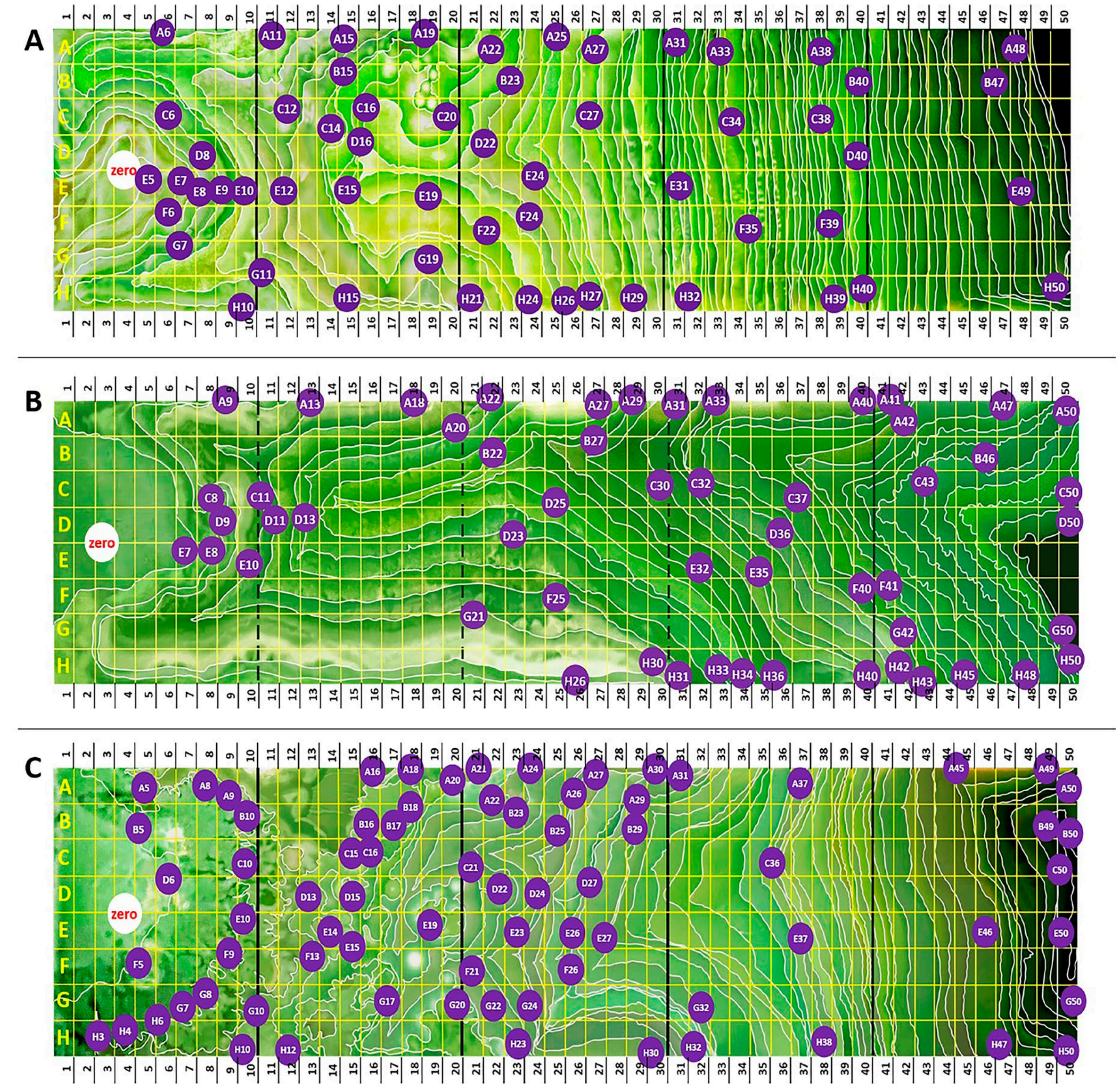

Figure S2. Topology of isolates selected for phenotypic and genetic analyses during the experimental adaptation of three *P. aeruginosa* lineages to increasing colistin concentrations. Three *P. aeruginosa* strains, including *Pa_ATCC* (upper panel), *Pa_Environment* (middle panel), and *Pa_MDR* (lower panel), were inoculated (point "zero") into the experimental plate containing an exponential gradient of colistin from 0 to 400 mg/L (see Figure 1 for more details). Each panel was arbitrarily divided into coordinates, and collected isolates were encoded accordingly. The black vertical lines on numbers 10, 20, 30, and 40 indicate the boundaries of the colistin bands of 2, 4, 40, and 400 mg/L, respectively. Each circle indicates an isolate and its coordinates in the experimental plate, where it was collected. The white lines delineate the bacteria growth front at different time points in the experiment.
