# Supplementary material for: Genetic Alternatives for Experimental Adaptation to Colistin in Three Pseudomonas aeruginosa Lineages
Source: Antibiotics (Basel). 2024 May 15;13(5):452. doi: 10.3390/antibiotics13050452 (PMC11117860; doi:10.3390/antibiotics13050452)
Supplement: Supplementary file 1 [file antibiotics-13-00452-s001.zip › Supplementary Table S1.pdf]

Table S1. Individual core gene alterations in the *Pa_ATCC* lineage, by colistin concentration band and time

Isolate		Day	Colistin		*phoQ*	*phoP*	*lpxL*/PA0011	*parR*	*nuoM*	*folK*	*dnaK*	*anr*	*sdhA*	*ppiD*	*hscA*	*pilJ*	*ppkA*
ID	Topology		Plate, mg/L	MIC, fold increase													
0	**zero**	**0**	**0**	**1**													
1	**E5**	**1**	**0**	**1**													
2	**F6**	**2**	**0**	**2**													
3	**E7**	**2**	**0**	**2**													
5	**H10**	**4**	**0**	**4**													
6	**G7**	**4**	**0**	**4**													
7	**E9**	**4**	**0**	**4**													
8	**D8**	**4**	**0**	**4**													
9	**C6**	**4**	**0**	**2**													
10	**A6**	**4**	**0**	**4**													
17	**E10**	**6**	**0**	**4**													
16	**G11**	**6**	**2**	**4**													
18	**A11**	**6**	**2**	**4**													
23	**H15**	**8**	**2**	**4**													
24	**E12**	**8**	**2**	**4**													
25	**C12**	**8**	**2**	**4**													
26	**A15**	**8**	**2**	**4**													
34	**G19**	**10**	**2**	**8**													
35	**C14**	**10**	**2**	**4**													
36	**B15**	**10**	**2**	**4**													
37	**A19**	**10**	**2**	**8**													
33	**H21**	**10**	**4**	**4**													
52	**E19**	**13**	**2**	**4**													
53	**E15**	**13**	**2**	**4**													
54	**D16**	**13**	**2**	**4**													
55	**C16**	**13**	**2**	**4**													
56	**C20**	**13**	**2**	**4**													
50	**H24**	**13**	**4**	**4**													
51	**F22**	**13**	**4**	**4**													
57	**A22**	**13**	**4**	**8**													

Isolate		Day	Colistin		*phoQ*	*phoP*	*lpxL*/PA0011	*parR*	*nuoM*	*folK*	*dnaK*	*anr*	*sdhA*	*ppiD*	*hscA*	*pilJ*	*ppkA*
ID	Topology		Plate, mg/L	MIC, fold increase													
62	**H26**	**15**	**4**	**8**													
63	**F24**	**15**	**4**	**4**													
64	**D22**	**15**	**4**	**4**													
65	**B23**	**15**	**4**	**4**													
71	**H27**	**17**	**4**	**4**													
72	**E24**	**17**	**4**	**4**													
73	**A25**	**17**	**4**	**4**													
82	**H29**	**21**	**4**	**4**													
84	**C27**	**21**	**4**	**4**													
85	**A27**	**21**	**4**	**4**													
94	**H32**	**27**	**40**	**4**													
95	**E31**	**27**	**40**	**4**													
96	**A31**	**27**	**40**	**8**													
99	**F35**	**33**	**40**	**4**													
101	**A33**	**33**	**40**	**4**													
104	**H39**	**42**	**40**	**4**													
105	**F39**	**42**	**40**	**4**													
106	**C38**	**42**	**40**	**8**													
107	**A38**	**42**	**40**	**4**													
108	**H40**	**47**	**40**	**4**													
109	**D40**	**47**	**40**	**4**													
110	**B40**	**47**	**40**	**4**													
113	**H50**	**60**	**400**	**4**													
114	**E49**	**60**	**400**	**4**													
116	**A48**	**61**	**400**	**4**													

Note. Fifty-five isolates of the Pa_ATCC lineage were collected from the experimental plate. Details are provided in Table 2 of the main text. Mutation variants are shown in the Supplementary Table S3.
