# Supplementary material for: Genetic Alternatives for Experimental Adaptation to Colistin in Three Pseudomonas aeruginosa Lineages
Source: Antibiotics (Basel). 2024 May 15;13(5):452. doi: 10.3390/antibiotics13050452 (PMC11117860; doi:10.3390/antibiotics13050452)
Supplement: Supplementary file 1 [file antibiotics-13-00452-s001.zip › Supplementary Table S2.pdf]

Table S2. Individual core gene alterations in the *Pa_MDR* lineage, by colistin concentration band and time

Isolate		Day	Colistin		*phoQ*	*pmrB*	*lpxL*/PA0011	262,402 bp (239 ORF)	*lasR*	*capD*	*wecA*	*mexB*	*spaQ*	*invA*	*pcpR*	*davD*	*hp*/PA0285	*rpoB*	*rpoA*	*morA*	*exsD*	*degS*
ID	Topology		Plate, mg/L	MIC, fold increase																		
0	**zero**	**0**	**0**	**1**																		
1	**F5**	**1**	**0**	**2**																		
2	**D6**	**1**	**0**	**2**																		
3	**B5**	**1**	**0**	**2**																		
4	**H3**	**2**	**0**	**2**																		
5	**H4**	**2**	**0**	**1**																		
6	**H6**	**2**	**0**	**2**																		
7	**G7**	**2**	**0**	**4**																		
8	**G8**	**2**	**0**	**1**																		
9	**F9**	**2**	**0**	**2**																		
11	**C10**	**2**	**0**	**4**																		
12	**B10**	**2**	**0**	**1**																		
13	**A9**	**2**	**0**	**2**																		
14	**A8**	**2**	**0**	**1**																		
15	**A5**	**2**	**0**	**1**																		
28	**H10**	**4**	**0**	**1**																		
29	**G10**	**4**	**0**	**1**																		
30	**F13**	**4**	**2**	**1**																		
31	**E14**	**4**	**2**	**1**																		
32	**D13**	**4**	**2**	**2**																		
33	**C15**	**4**	**2**	**1**																		
34	**B16**	**4**	**2**	**1**																		
35	**A16**	**4**	**2**	**0.5**																		
36	**H12**	**5**	**2**	**1**																		
37	**E15**	**5**	**2**	**2**																		

Isolate		Day	Colistin		*phoQ*	*pmrB*	*lpxL*/PA0011	262,402 bp (239 ORF)	*lasR*	*capD*	*wecA*	*mexB*	*spaQ*	*invA*	*pcpR*	*davD*	*hp*/PA0285	*rpoB*	*rpoA*	*morA*	*exsD*	*degS*
ID	Topology		Plate, mg/L	MIC, fold increase																		
38	**D15**	**5**	**2**	**1**																		
39	**C16**	**5**	**2**	**1**																		
40	**B17**	**5**	**2**	**1**																		
41	**A18**	**5**	**2**	**1**																		
42	**B18**	**5**	**2**	**0.5**																		
52	**G20**	**7**	**2**	**1**																		
53	**G17**	**7**	**2**	**1**																		
54	**E19**	**7**	**2**	**1**																		
56	**A20**	**7**	**2**	**0.5**																		
57	**A21**	**7**	**4**	**2**																		
58	**A22**	**7**	**4**	**1**																		
66	**H23**	**9**	**4**	**2**																		
67	**G22**	**9**	**4**	**2**																		
68	**F21**	**9**	**4**	**1**																		
69	**C21**	**9**	**4**	**1**																		
70	**D22**	**9**	**4**	**4**																		
71	**B23**	**9**	**4**	**4**																		
72	**A24**	**9**	**4**	**4**																		
85	**H30**	**12**	**4**	**4**																		
86	**G24**	**12**	**4**	**8**																		
87	**E23**	**12**	**4**	**1**																		
88	**D24**	**12**	**4**	**4**																		
89	**B25**	**12**	**4**	**1**																		
90	**A26**	**12**	**4**	**2**																		
91	**A27**	**12**	**4**	**1**																		
107	**A30**	**15**	**4**	**8**																		
109	**F26**	**16**	**4**	**2**																		

Isolate		Day	Colistin		*phoQ*	*pmrB*	*lpxL*/PA0011	262,402 bp (239 ORF)	*lasR*	*capD*	*wecA*	*mexB*	*spaQ*	*invA*	*pcpR*	*davD*	*hp*/PA0285	*rpoB*	*rpoA*	*morA*	*exsD*	*degS*
ID	Topology		Plate, mg/L	MIC, fold increase																		
110	**E26**	**16**	**4**	**1**																		
111	**D27**	**16**	**4**	**2**																		
112	**A29**	**16**	**4**	**4**																		
108	**A31**	**16**	**40**	**8**																		
119	**E27**	**18**	**4**	**2**																		
121	**B29**	**18**	**4**	**1**																		
118	**G32**	**18**	**40**	**32**																		
122	**A32**	**18**	**40**	**4**																		
144	**H38**	**27**	**40**	**4**																		
145	**E37**	**27**	**40**	**4**																		
146	**C36**	**27**	**40**	**2**																		
147	**A37**	**27**	**40**	**32**																		
159	**H47**	**42**	**400**	**32**																		
160	**E46**	**42**	**400**	**8**																		
161	**A45**	**42**	**400**	**4**																		
162	**H50**	**44**	**400**	**8**																		
163	**A49**	**44**	**400**	**32**																		
164	**A50**	**46**	**400**	**32**																		
169	**G50**	**47**	**400**	**32**																		
170	**E50**	**47**	**400**	**32**																		
171	**C50**	**47**	**400**	**64**																		
172	**B49**	**47**	**400**	**8**																		
173	**B50**	**47**	**400**	**64**																		
