# Supplementary material for: Genetic Alternatives for Experimental Adaptation to Colistin in Three Pseudomonas aeruginosa Lineages
Source: Antibiotics (Basel). 2024 May 15;13(5):452. doi: 10.3390/antibiotics13050452 (PMC11117860; doi:10.3390/antibiotics13050452)
Supplement: Supplementary file 1 [file antibiotics-13-00452-s001.zip › Supplementary Table S3.pdf]

Table S3. Sequence alterations in general metabolism genes not directly related to colistin resistance among three experimental lineages during adaptation to increasing colistin concentrations.

Gene	Sequence alteration	
	DNA	AA
***Pa_ATCC* lineage**		
parR	G466→A, missense	E156K
nuoM	del-TTC-427-429	del-F143
folK	T440→C , missense	L147P
dnaK	A517→G, missense	T173A
anr	del-GCT-465-467	del-L156
sdhA	A854→G , missense	D285G
ppiD	ins-G-1287	G429fs, incorrect protein
hscA	A384→C, missense	E128D
pilJ	del-A-1754	S584fs, incorrect protein
ppkA	A1126→G, missense	S376G
***Pa_Environment* lineage**		
cysL	G746→A, missense	R249H
prs	T143→C, missense	V48A
brnQ	del-12bp-566-577	del-F189-G192
speE	T836→C, missense	V279A
fha1	ins-ACAGCC-849	ins-Q284-P285
***hp/*PA2072**	del-13bp-908-920	E302fs, incorrect protein
***hp/*PA2117**	G326→A, missense	R109H
tetC	G332→A, missense	R111H
oprH	ins-G-192	G64fs, incorrect protein
pykF	G676→A, missense	A226T
***Pa_MDR* lineage**		
lasR	G502→T, nonsense	E168*, incomplete protein
capD	del-T-360	A119fs, incorrect protein
wecA	C313→T, missense	H105Y
mexB	C2795→T , missense	T932M
spaQ	T122→C , missense	L41P
invA	ins-G-1726	G575fs, incorrect protein
pcpR	G430→T , missense	A144S
davD	TGA1450-1452→CTC, stop codon loss	incorrect protein
***hp/*PA0285**	G1516→A, missense	E506K
rpoB	del-24bp-850-873	del-K284-E291
rpoA	ins-CTGGTC-604	ins-L202-V203
morA	A3121→C, missense	T1041P
exsD	G64→A, missense	V22M
degS	T692→A, missense	L231Q
Note. AA, amino acid		
